# Strengthening ethical community engagement in contemporary Malawi

**DOI:** 10.12688/wellcomeopenres.14793.2

**Published:** 2019-03-18

**Authors:** Deborah Nyirenda, Kate Gooding, Rodrick Sambakunsi, Linley Seyama, Joseph Mfutso-Bengo, Lucinda Manda Taylor, Stephen B. Gordon, Michael Parker

**Affiliations:** 1 Malawi Liverpool Wellcome Trust Clinical Research Programme, Blantyre, Malawi; 2 Liverpool School of Tropical Medicine, Liverpool, UK; 3 University of Malawi College of Medicine, Blantyre, Malawi; 4 Johns Hopkins Project, Blantyre, Malawi; 5 The Centre for Bioethics in Eastern and Southern Africa (CEBESA), University of Malawi, College of Medicine, Blantyre, Malawi; 6 The Ethox Centre/ Wellcome Centre for Ethics and Humanities, University of Oxford, Oxford, UK

**Keywords:** Community engagement, health research, research ethics, Malawi

## Abstract

Although community engagement is increasingly promoted in global health research to improve ethical research practice, there is sometimes a disconnect between the broader moral ambitions for community engagement in the literature and guidelines on the one hand and its rather narrower practical application in health research on the other. In practice, less attention is paid to engaging communities for the ‘intrinsic’ value of showing respect and ensuring inclusive participation of community partners in research design. Rather, more attention is paid to the use of community engagement for ‘instrumental’ purposes to improve community understanding of research and ensure successful study implementation. Against this backdrop, we reviewed the literature and engaged various research stakeholders at a workshop to discuss ways of strengthening ethical engagement of communities and to develop context-relevant guidelines for community engagement in health research in Malawi.

## Disclaimer

The views expressed in this article are those of the author(s). Publication in Wellcome Open Research does not imply endorsement.

## Introduction

Guidelines and literature on community engagement emphasise participatory processes of engagement as a way of showing appropriate respect to communities and as a means of building sustainable community trust and equitable collaborative partnerships between researchers and community stakeholders. For instance, ethics guidelines from the Council of International Organisations of Medical Sciences (CIOMs) state that:

*'researchers, sponsors, health authorities and relevant institutions should engage potential participants and communities in a meaningful participatory process that involves them in an early and sustained manner in the design, development and implementation, implementation of informed consent processes and monitoring of research and in the dissemination of its results*' ^1^.

Similar themes of participatory governance and involving communities as partners rather than research participants are found in guidance on ensuring effective community engagement, educating researchers and evaluating research that engages with the community. For instance, Ahmed and Palermo’s (2010) framework for reviewing applications for research involving communities includes values such as mutual understanding of the meaning of community engagement, strong partnerships, equitable sharing of power and responsibilities, inclusion of diverse perspectives, relevant research goals, ensuring mutual benefit for all partners, capacity building for both partners, equal respect, continuous communication, and transparent monitoring and evaluation. In order to measure levels of community participation in research, others have developed evaluation tools to capture levels of community participation throughout the research cycle. For example, Khodyakov’s (2012) Community Engagement in Research Index measures levels of community participation throughout the research cycle. The Index identifies 12 specific activities including involvement in grant proposal writing, study design, study implementation, data collection and analysis and dissemination of results in journal articles and conferences, and measures community participation against each activity by indicating whether the community was actively engaged, consulted or did not participate ^2^. Standards developed by research funders also support participatory governance. For example, the UK National Institute for Health Research standards for public involvement in research include inclusive opportunities, working together, support and learning, working together, communications, impact and governance ^3^.

### The practical realities: community engagement as a useful tool

While guidelines emphasise participatory decision-making throughout the research cycle, in practice community engagement is often perceived by researchers in narrower, more ‘instrumental’ terms, for example as a means of clearing concerns, satisfying funders’ requirements, increasing visibility, and maximizing study participation and acceptability, particularly in low resource settings ^4, 5^. Many publications report use of community sensitization meetings, media, and involvement of community leaders, community representatives and other stakeholders to increase knowledge of medical research, improve study acceptability and support trial implementation, rather than engaging communities in participatory processes throughout research design and implementation ^6– 11^.

This approach to community engagement is understandable to some extent, given the time limitations and other pressures researchers and research institutes face in conducting research. Specifically, grant writing requires presentation of detailed methodology before funding is available for community consultation. Pre-award funding is also unusual and limited in both monetary value and time. In addition to these concerns, higher levels of engagement are sometimes seen as unfeasible where communities are judged to lack adequate education and understanding of research to provide input without the allocation of significant time and effort by researchers to educational and other activities. For instance, several studies have reported challenges in engaging community members as collaborative partners through community advisory boards (CABs) or community advisory groups (CAGs). These studies have reported conflicting roles of CABs, which are asked to provide input to protocol development and minimize harm but also to advance research goals by facilitating participant recruitment ^12– 14^. Other challenges include limited understanding of health research and monetary expectations among CAB members, dependence on researchers for finances, and lack of authority to influence decisions concerning research ^15– 18^.

Overall, this implies that while community engagement is promoted in guidelines and literature relating to global health research as a means to improve protection, mutual benefits, legitimacy and shared decision making ^19, 20^, there are also challenges in implementation of community engagement at the levels recommended in ethics guidance. Given the practical challenges, there are unanswered questions about what counts as an appropriate and acceptable approach to community engagement in the non-ideal situations in which research is often planned and conducted. What is required when the perfect is not achievable? Further guidance and evidence are therefore needed to support community engagement in health research in low literacy settings. In order to promote discussion on this topic in our setting, we organised a stakeholder workshop to gather ideas on two issues as a basis for improving community engagement guidance and practice in Malawi: 1) appropriate levels of engagement, and 2) consideration of engagement within ethics review.

A range of community engagement activities currently take place in Malawi, including community or stakeholder meetings, and use of radio, film, TV programmes and other information or education materials. These activities tend to focus on one-way flow of information from researchers to communities, and have primarily been used by researchers to improve understanding of research and support informed consent. Some publications from Malawi have examined community responses in health research and developed benchmarks for community engagement ^21– 24^. However, there are no regulatory requirements to comply with these benchmarks for community engagement. In addition, while CIOMS guidelines are used during ethics review, community engagement is not a requirement for all studies conducted in Malawi. 

## Methods

The discussion in this paper was drawn from a participatory workshop on ethical issues in health research which took place in Zomba, Malawi from 23 to 24 ^th^ May, 2018. A total of 50 participants attended the workshop, including delegates from research institutions, Research Ethics Committees (RECs), local and national government and the Pharmacy Medicines and Poisons Board of Malawi. This workshop was used to stimulate discussions on how higher levels of community engagement can be attained in a low literacy, low income setting such as Malawi. We presented ethical guidance from CIOMS on community engagement and frameworks that have been developed to systematically review or assess community engagement in research. Afterwards, delegates were divided into four groups to discuss the level of engagement to aspire for in Malawi, including appropriate stages for engagement in the research process and how the level of engagement should vary between research projects, and whether and how RECs should consider community engagement when reviewing research proposals. Discussions from the small groups were documented on flip chart papers and presented in a plenary session. Following this, authors of this paper consolidated the discussions from the four groups and presented a summary and some action points to the whole group. The following is a summary of the group discussions and action points to strengthen ethical engagement in Malawi. 

## Results and discussion

### Strengthening ethical community engagement in Malawi

Workshop participants agreed that community engagement should be incorporated in health research activities and that this should involve more than seeking letters of support from stakeholders such as hospital directors, District Health Officers or simply ‘informing’ communities about the research. According to Arnstein’s ladder of citizen participation, this type of engagement where stakeholders are engaged to get public support is considered as non-participation ^25^.

While there was broad consensus at the stakeholder workshop about the importance of engaging communities in health research beyond just sharing information, several concerns were raised in relation to the feasibility of engaging community members as genuinely equitable partners and involve them in decision making. These concerns included challenges in responding to complex community needs that may not be in line with research priorities. There were also concerns about identifying relevant community representatives in hospital or laboratory based studies or research involving vulnerable populations. Participants also identified funding limitations, time constraints, low literacy levels among communities as well as lack of guidance on community engagement as hindering participatory engagement.

**Figure 1.**
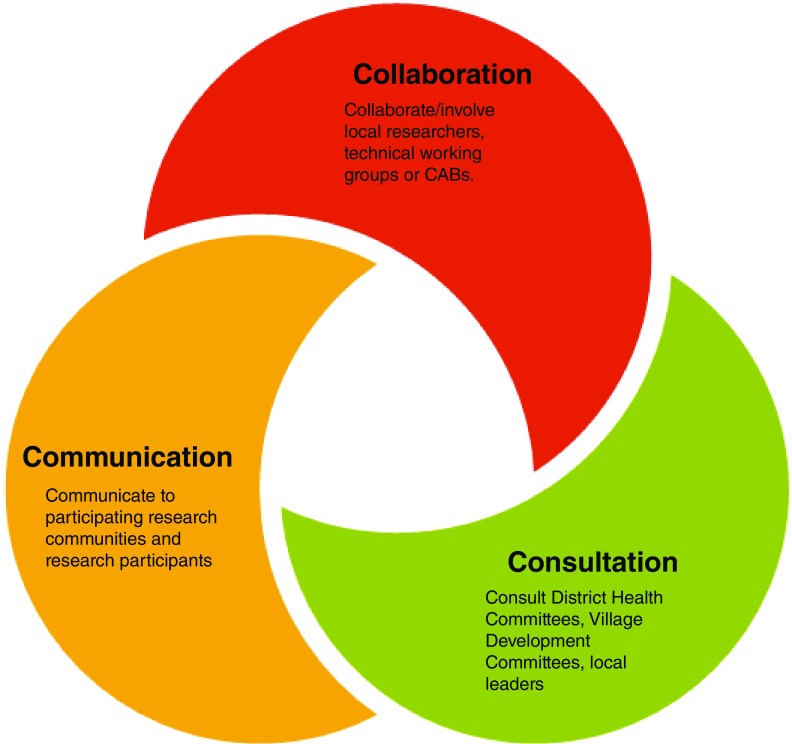
The 3 C’s model of participatory community engagement.

Taking into consideration these concerns, workshop participants offered alternative ways in which community engagement activities in a low literacy setting can move up the ladder of participation from simply information sharing to more collaborative partnerships. In the following, we propose an innovative model to provide guidance on how participatory community engagement can be attained throughout the research process. Our proposed model has three steps or 3C’s of: ‘collaboration’, ‘consultation’ and ‘communication’. This model however does not aim to rank the different types of engagement in order of priority. In addition, we also used Dickert’s (2015) ethical goals of consulting communities to give examples of how we can move up the ladder of participation to enhance protection, benefits, legitimacy and shared responsibility. By referring to three themes of collaboration, consultation and communication, we use insights from workshop discussions to describe how community engagement can move up the ladder of participation with different community groups (See Figure 1).

***1) Enhancing collaborative partnerships.*** In order to achieve genuine collaborative partnerships, workshop participants suggested that various community representatives should be engaged as early as possible and throughout study implementation. Since engagement of lay community members during protocol development is seen as unfeasible due to low scientific literacy, local researchers who have an understanding of scientific research, government technical working groups, or community advisory boards (CAB) should be engaged to ensure that the research is relevant to the community and that participating research communities are protected from harm. The CAB should include people who have been adequately empowered to scrutinize research proposals. For instance, one workshop participant indicated that they present research protocols to an institutional CAB for input. This CAB includes representatives from different community groups such as school communities, religious leaders, activists, service providers, government and many more. Even though this CAB may not be effective in representing typical views of local community members, they provide advice aimed at enhancing protection and maximising benefits for participating research communities. This CAB strategy therefore overcomes some of the challenges pertaining to power imbalances between researchers and community representatives’ due to differences in literacy, and enhances legitimacy and shared responsibility in research design.

***2) Community consultations.*** At a community level, to achieve effective community consultations, workshop participants indicated that existing local community representatives or stakeholders such as District Health Committees and Village Development Committees should be consulted to identify social risks of research and minimize harm. While local stakeholders such as researchers may be engaged as collaborators to give input during protocol development, views of lay community members on the study design must also be respected. Although most people in participating research communities are illiterate and poor, they have a better understanding of their community needs as well as social norms than researchers. As such, these community stakeholders should be consulted to provide input on the research design to ensure that research is relevant to the context. An example was given concerning a study that required women to stop breast-feeding babies at six months. The researchers presented the research design to community leaders and they advised that most women would not follow the research procedures and stop breastfeeding their babies as advised. By consulting the community leaders, the risks of compromising the quality of data was minimised and research participants were protected from harm.

***3) Communication with participating research communities and research participants.*** In addition to phases one and two above, community members should be informed about the research to improve understanding of research objectives. Community meetings, radio and other information and educational materials could therefore be used to ensure regular communication with communities. In addition, research participants, participating research communities as well as the other research stakeholders must be informed about the study results. The 3 C’s model of participatory community engagement can therefore be applied iteratively at different stages of the research to respond to community needs and ensure ethical conduct of research.

## Conclusion

International ethical guidelines for health research promote participatory processes of engaging communities in research. Community engagement practices however do not always reflect the ideals of participatory governance promoted in the literature particularly in low literacy settings. We used a workshop to engage various research stakeholders in Malawi to discuss ways of improving community engagement practices in Malawi. This paper presents our proposed model of community engagement to guide researchers and regulators as they strive to increase community participation in health research. Our model presents three elements that would increase participatory community engagement in health research namely: collaboration, consultation and communication. The novelty of this model is its emphasis on participatory community engagement with existing stakeholder groups that can be engaged from the onset of research. Our wish was to present a workable strategy that can be adopted by researchers in current conditions in Malawi. Finding the right balance of activities may help to strengthen ethical community engagement.

Workshop participants also indicated a number of strategies and policy measures that could support and encourage researchers in adopting this model of engagement. Particular activities include development of guidelines and checklists for planning community engagement, training in community engagement for researchers, field workers and community representatives, attention to community engagement within national policy on health research, and inclusion of a section on community engagement in protocols and research ethics committee review guidelines. In order to ensure adherence to ethical guidelines on community engagement and minimize tokenistic engagement, ethics review committees need to develop measures to audit community engagement activities as part of quality assurance. We plan to publish these guidelines and checklists for community engagement in the future.

## Data availability

No data are associated with this article.
