# Supplementary material for: Strengthening ethical community engagement in contemporary Malawi
Source: Wellcome Open Res. 2019 Mar 18;3:115. Originally published 2018 Sep 18. [Version 2] doi: 10.12688/wellcomeopenres.14793.2 (PMC6259484; doi:10.12688/wellcomeopenres.14793.2)
Supplement: Peer review history

# Peer review: Reviewer response for version 2

**DOI:** 10.21956/wellcomeopenres.16527.r35083

**Published:** 2019-04-04

**Authors:** Graham Lindegger

**Affiliations:** 1 University of KwaZulu-Natal (UKZN), Pietermaritzburg, South Africa

I have read the revised manuscript. I am satisfied that the manuscript sufficiently addresses my concerns to be accepted for indexing as is.

I have read this submission. I believe that I have an appropriate level of expertise to confirm that it is of an acceptable scientific standard.

# Peer review: Reviewer response for version 1

**DOI:** 10.21956/wellcomeopenres.16120.r34158

**Published:** 2018-12-18

**Authors:** Graham Lindegger

**Affiliations:** 1 University of KwaZulu-Natal (UKZN), Pietermaritzburg, South Africa

Overall, I found this manuscript very well written and potentially making an important contribution to the field. The manuscript focuses on how best to achieve better quality community engagement in research. One of the greatest strengths of the article lies in the argument for the distinction between intrinsic/ethical engagement with communities and instrumental/practical engagement. The article presents a good argument for the way in which community engagement can be reduced to purely practical considerations. While looking to enhance genuine community engagement, it also recognizes the challenge in this process. I would recommend that the article is accepted for publication following minor changes.

*First*, while the "three C' model is very useful it was not altogether clear what the distinction was between 1) and 2).

*Second*, under the section on "Enhancing collaborative partnerships" there is mention of "different levels" but it is not clear what this mean?

*Third,* I wondered whether all three aspects (or 'C's) could not apply at different stages of research.

*Fourth*, there seems little mention of the implications for ethical review process. A slightly fuller development of this would add to the value of the manuscript.

I have read this submission. I believe that I have an appropriate level of expertise to confirm that it is of an acceptable scientific standard, however I have significant reservations, as outlined above.

# Response

**Published:** 2019-02-22

**Authors:** Deborah Nyirenda

**Affiliations:** Malawi Liverpool Wellcome Trust Clinical Research Programme, Malawi

Thank you so much for your valuable feedback on our paper. We have revised the paper to incorporate your comments. We are however limited to give insights on implications for ethical review processes based on the workshop discussions.

# Peer review: Reviewer response for version 1

**DOI:** 10.21956/wellcomeopenres.16120.r34186

**Published:** 2018-11-26

**Authors:** Sarah Banks

**Affiliations:** 1 Department of Sociology, Durham University, Durham, UK

This letter discusses means of improving the ethical basis of community-engaged research in Malawi, based on the findings of a workshop. This is an important topic and the letter identifies a range of issues in a clear and accessible way. A model is suggested based on collaboration, consultation and communication. The model assumes 'outside' researchers needing to engage local stakeholders.   A more 'radical' model might include research starting from community concerns in which professional researchers are partners.

I have read this submission. I believe that I have an appropriate level of expertise to confirm that it is of an acceptable scientific standard.

# Peer review: Reviewer response for version 1

**DOI:** 10.21956/wellcomeopenres.16120.r34020

**Published:** 2018-10-15

**Authors:** Christine Grady

**Affiliations:** 1 Department of Bioethics, National Institutes of Health (NIH), Bethesda, MD, USA

Thank you for the opportunity to review this open letter.  Community engagement is an important feature of ethical clinical research.  I agree with the authors that the practice of community engagement is often instrumental, and researchers could pay more attention to the intrinsic values of engaging communities.  The authors write about a workshop they held to discuss ways of strengthening community engagement, especially in Malawi.  The background rationale was clear and compelling, they used the literature appropriately.  The report of their workshop process and results was very good.  Their proposed model for strengthening community engagement practices is useful and appreciated.

I have read this submission. I believe that I have an appropriate level of expertise to confirm that it is of an acceptable scientific standard.
